# Arthroscopic skills assessment and use of box model for training in arthroscopic surgery using Sawbones – “FAST” workstation

**DOI:** 10.1051/sicotj/2016024

**Published:** 2016-11-01

**Authors:** Saumitra Goyal, Mohamed Abdel Radi, Islam Karam-allah Ramadan, Hatem Galal Said

**Affiliations:** 1 Orthopaedics Department, Faculty of Medicine, Assiut University Hospital Assiut 71515 Egypt; 2 G.G. Medical Institute and Research Centre 106/2 Sanjay Place Agra 282004 India; 3 Arthroscopy & Sports Injuries Unit, Orthopaedics Department, Faculty of Medicine, Assiut University Assiut 71515 Egypt

**Keywords:** Athroscopy skills, Task performance, Assessment, Surgical training and benchmark, FAST module

## Abstract

*Purpose*: Arthroscopic skills training outside the operative room may decrease risks and errors by trainee surgeons. There is a need of simple objective method for evaluating proficiency and skill of arthroscopy trainees using simple bench model of arthroscopic simulator. The aim of this study is to correlate motor task performance to level of prior arthroscopic experience and establish benchmarks for training modules.

*Methods*: Twenty orthopaedic surgeons performed a set of tasks to assess a) arthroscopic triangulation, b) navigation, c) object handling and d) meniscus trimming using SAWBONES “FAST” arthroscopy skills workstation. Time to completion and the errors were computed. The subjects were divided into four levels; “Novice”, “Beginner”, “Intermediate” and “Advanced” based on previous arthroscopy experience, for analyses of performance.

*Results*: The task performance under transparent dome was not related to experience of the surgeon unlike opaque dome, highlighting the importance of hand-eye co-ordination required in arthroscopy. Median time to completion for each task improved as the level of experience increased and this was found to be statistically significant (*p* < .05) e.g. time for maze navigation (Novice – 166 s, Beginner – 135.5 s, Intermediate – 100 s, Advance – 97.5 s) and the similar results for all tasks. Majority (>85%) of subjects across all the levels reported improvement in performance with sequential tasks.

*Conclusion*: Use of the arthroscope requires visuo-spatial coordination which is a skill that develops with practice. This simple box model can reliably differentiate the arthroscopic skills based on experience and can be used to monitor progression of skills of trainees in institutions.

## Introduction

With advancement in minimally invasive surgical interventions, arthroscopic surgery is now one of the most common procedures performed in modern orthopaedics [1]. Unlike open surgery, arthroscopic procedures involve 2-D image projection of a three-dimensional operative field requiring more technical dexterity and visuo-spatial coordination [2–4]. Proficiency in arthroscopy has a steep learning curve and before embarking on clinical arthroscopy, surgeons should be competent in handling instruments and learn the basic skills of arthroscopy [3–5]. There is a need for out of operating room practice of these skills so as to decrease errors in instrument handling, risks of iatrogenic injury, financial burden and operative time during the initial phase of practice. This is further limited by introduction of restricted working hours for residents across Europe and North America affecting *hands-on* training [2, 3, 6–10]. Arthroscopic simulators, including cadaver and bench models. More recently, computerized virtual reality simulators have evolved over time to teach psychomotor skills of arthroscopic surgery [2–5, 7–9, 11–24]. Cadaver models and virtual reality simulators are resource demanding whereas the bench models are easy to set up, simulate realistic environment and have shown development of motor skills and technical training [2, 7–9, 11, 20, 22–24]. Simulated tasks involving *image tracking*, *triangulation and probing* and *handling of basic tools* like shaver, punch and grasper can replicate basic skills of arthroscopic surgery. Performance in these tasks has been shown to match clinical experience however, there is need of *structured modules* that can be applied in training curriculum to establish the proficiency of trainees [2, 3, 25–31]. Collaborative efforts of American orthopaedic and arthroscopy associations have proposed one such module for arthroscopic skills training; the “*FAST* program” [29].

The module uses a simple box-trainer type arthroscopic simulator and we hypothesized that more advance surgeons would perform better in *tasks* based on experience. Hence, this study was designed and conducted to test the usefulness of this model to assess performance based on clinical experience and obtain data to design guidelines for future arthroscopic training.

## Methodology

### Study design and subjects

This prospective cohort study was conducted in the sports medicine and arthroscopy unit of a University Hospital during the period of September to October 2014. The study included 20 orthopaedic surgeons of various levels of arthroscopic experience including professors, lecturers, assistant lecturer grade surgeons, trainees including year 1–3 orthopaedic residents. None of the subjects had previous exposure to practice on the test workstation. They were given written instruction and video demonstration of the tasks and consent to participate in the study was obtained.

The 20 orthopaedic surgeons in the study consisted of 12 participants nonproficient in arthroscopic surgery (7 residents, 3 clinical fellows, 2 trauma surgeons) and 8 arthroscopy specialists (5 assistant lecturers or lecturers, 3 professors) and their demographic details were obtained as presented in Table 1.

**Table 1.** Participants profile and arthroscopic experience of subgroups.

Group characteristics	Novice	Beginner	Intermediate	Advance
Independent procedures	None	<50	50–100	>200
Number (% of total)	9 (45%)	4 (20%)	3 (15%)	4 (20%)
Mean age (years, ±*SD*)	32.3 (±6.1)	33.5 (±3.3)	32 (±2.6)	44 (±6.9)
Mean years of orthopaedic practice	3	8	7	19.5
Clinical arthroscopy practice (years)	None	<2	2–4	>4
Knee arthroscopy exposure*	None	All	All	All
Shoulder arthroscopy exposure*	None	1/4	2/3	All
Independent surgeon*	None	2/4	All	All

* Out of the total number in the group.

Arthroscopy experience was quantified as the number of independently performed procedures involving basic knee arthroscopic surgery. The subjects were divided into four groups as follows: (1) Novice – no exposure to arthroscopy, (2) Beginner – less than 50 cases independently performed, (3) Intermediate – 50–100 cases performed independently and (4) Advance – more than 200 cases as independent surgeon.

### Equipment and task specification

**Figure 1.**
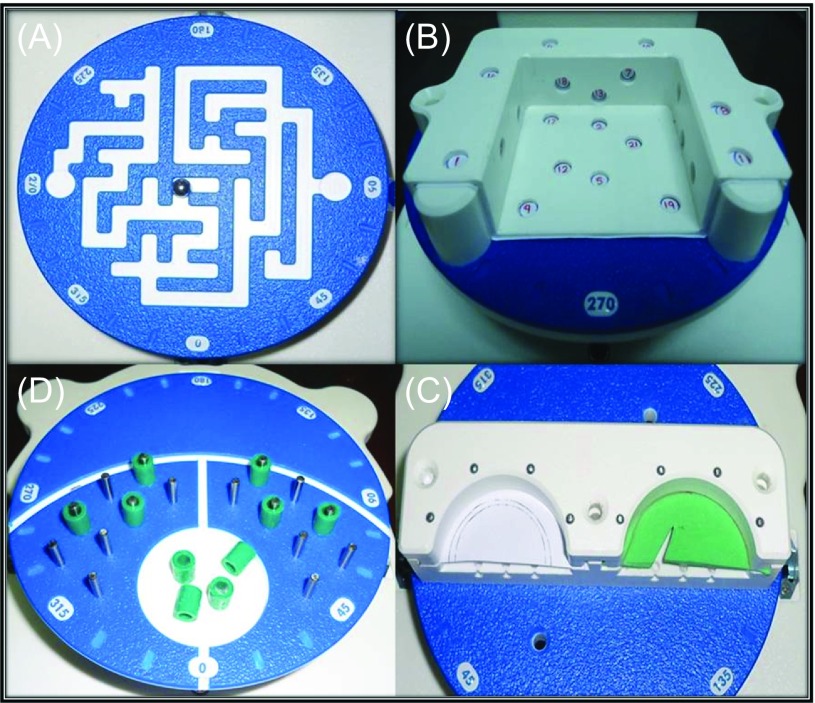
Modules for “FAST” bench workstation. (A) Maze navigation, (B) number probing, (C) object handling-vertical pillars and (D) meniscus stations.

The study was conducted using a bench-type workstation with geometrical objects placed in a three-dimensional environment for the use of basic arthroscopy instruments. The *box-trainer* was developed by “SAWBONES” (Pacific Research Laboratories) in collaboration with the “FAST” (Fundamentals of Arthroscopic Surgery Training) program. The “FAST” program consists of modules for sequential proficiency in basic and advanced motor skills proposed by combined efforts of AANA (Arthroscopy Association of North America), AAOS (American Academy of Orthopaedic Surgeons) and ABOS (American Board of Orthopaedic Surgery) to become a part of structured curriculum and training in arthroscopic surgery [30]. The workstation consists of multiple detachable rotating platforms like maze navigation, randomly arranged number probing station, horizontal and vertical pillar stands for object placement and extraction and meniscus platform for simulating basic and interventional knee arthroscopy skills (Figure 1).

**Figure 2.**
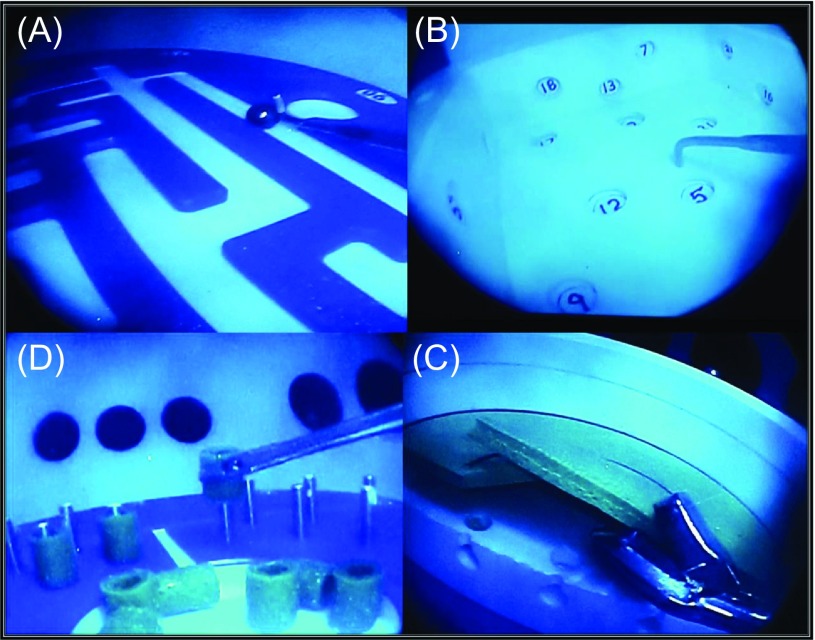
Opaque dome (OD) modules for “FAST” bench workstation. (A) Maze navigation, (B) number probing, (C) object handling-vertical pillars and (D) meniscus stations.

Competency in scope navigation, triangulation, probing and handling of objects within three-dimensional space and partial menisectomy were chosen from the modules to assess basic arthroscopy skills. The tasks used as surrogate for these skills were: (1) maze navigation, (2) number probing, (3) object extraction and insertion and (4) partial menisectomy of medial and lateral meniscus (Table 2, Figure 2).

**Table 2.** Description of basic arthroscopy skills and surrogate tasks on the “FAST” workstation.

Skill	Description of skill	Task surrogate
Diagnostic arthroscopy	Scope navigation (telescoping, periscoping), Triangulation, Probing	Maze navigation task, Number probing task
Tissue handling	Probing, Simultaneous image tracking and instrument handling, Bimanual dexterity, Grasp and release	Loose body extraction and insertion task – vertical and horizontal pillars
Partial menisectomy	Meniscus resection and balancing, Use of instrument – punch	Medial and Lateral meniscus partial menisectomy task

After the instructional video on task performance, participants were asked to perform all four tasks under a transparent dome (TD) with direct vision and then under the opaque dome (OD) with the arthroscope using the portals representative for antero-medial and antero-lateral arthroscopy portals for the right knee. The equipment was standardized for all in terms of position of platform and instruments used which consisted of a standard 30° arthroscope with light source and camera, LCD monitor, 5 mm probe and straight grasper for object handling and a straight cutting punch for menisectomy.

### Individual task protocols (Figure 2)

1. Maze navigation: Subjects completed the task of probing a steel ball (3 mm diameter) through the maze without skipping channels or dropping the ball which was counted as an error. The platform was fixed at 0° rotation.
2. Number probing: In this task the participants were asked to sequentially probe numbers 1–21 randomly arranged on a predesigned perforated platform which fixed at 270° front facing and the time to complete was computed under both TD and OD.
3. Object handling: This task was considered representative of tissue handling skill and ability to use instruments with bimanual dexterity. The task required placement and retrieval of 10 tubular cylinders of each 12 mm size into two different spatial arrangements of horizontal and vertical pillars. Dropping the object was counted as an error and the time to complete all 10 objects in the task was taken as the end point.
4. Partial menisectomy: The specially designed platform for representation of meniscus was rotated to 45° on either side to represent medial (MM) and lateral meniscus (LM) for the right knee. A 1 mm standard density foam material was tested and agreed upon by three arthroscopy experts to represent the feel of cutting meniscus tissue by punch. A semicircular meniscus was fashioned out of the foam and a mark was made to represent red-white zone with a radial tear. The participants were then asked to trim the meniscus to within the marked zone with precision first under the transparent then under opaque the dome. Time to complete task and the precision of “menisectomy” were noted.

Additional platforms for shoulder arthroscopy tasks like anchor placement, suture passage and knot tying were not used in this study.

### Assessment parameters and analysis

Time for task completion and number of errors were used as objective assessor of proficiency and discriminant validity of construct. For each error the participants were penalized 5 seconds on the clock but allowed to complete the task. Subjective assessment of steadiness of scope, simultaneous image tracking and instrument handling, bimanual dexterity and face validity was done by feedback questionnaire from the participant and assessment by an unbiased observer of performance using a Likert scale from 1 to 5 (1-very easy or very good/2-easy or good/3-not so difficult or not so good/4-difficult or poor/5-very difficult or very poor). Performance measured as time for task completion under transparent and opaque dome comparisons within and between groups was estimated using the non-parametric statistical tests (Mann-Whitney U, Kruskal-Wallis H test). All analysis was done using commercial statistical package SPSS (Version 16, SPSS Inc, Chicago, IL) for MS Windows. A *p-value ≤ 0.05* was considered statistically significant during the analysis.

## Results

The mean time taken for completion of tasks performed under the transparent dome and opaque dome is presented in Table 3 and the comparison of performance between groups with improvement of performance based on experience is presented in Table 4.

**Table 3.** Mean times (±*SD*) for completion of task for different sub-groups under transparent dome (TD) and opaque (OD) domes.

	Maze navigation		Number probing		Object retrieval		Object placement		Partial menisectomy	
	TD	OD	TD	OD	TD	OD	TD	OD	TD	OD
Novice	66.5 (±10.4)	179.7 (±71.4)	128.1 (±32.7)	657.0 (±195.7)	108.2 (±23.4)	388.3 (±165.8)	230.6 (±46.8)	626.2 (±150.3)	120.4 (±32.3)	364.1 (±156.0)
Beginner	56.0 (±8.9)	130.0 (±32.0)	134.0 (±29.5)	333.0 (±166.9)	90.7 (±11.8)	247.7 (±45.3)	162.7 (±30.8)	433.7 (±116.2)	100.7 (±22.9)	208.7 (±29.6)
Intermediate	53.6 (±8.1)	104.3 (±18.8)	141.0 (±19.0)	267.0 (±119.3)	79.3 (±2.5)	162.3 (±28.9)	141.3 (±30.0)	269.0 (±46.5)	110.3 (±48.3)	142.6 (±110.3)
Advance	57.2 (±10.3)	100.7 (±23.8)	127.2 (±36.7)	258.2 (±71.3)	87.2 (±11.2)	146.7 (±36.1)	159.2 (±43.3)	276.2 (±49.2)	56.75 (±10.2)	85.7 (±39.2)
*p value* (*ANOVA*) – *between groups*	0.152	0.027	0.924	0.001	0.084	0.011	0.090	<0.001	0.052	0.005

**Table 4.** Difference in performance under the opaque dome (OD) as measured by percent improvement between subsequent groups with higher level of expertise of subjects.

Task in OD	Novice time (min)	Beginner time (min)	Intermediate time (min)	Advance time (min)	Novice to Beginner difference		Beginner to Intermediate difference		Intermediate to Advance difference	
	Median (range)	Median (range)	Median (range)	Median (range)	% improv	*p* value	% improv	*p* value	% improv	*p* value
Maze	166 (124–360)	135.5 (86–163)	100 (88–125)	97.5 (78–130)	75	0.273	67	0.593	33	0.285
Probing	684 (325–960)	254.5 (240–583)	228 (172–401)	260 (187–326)	100	0.068	67	1.00	67	1.00
Object extraction	319 (227–679)	240 (202–309)	171 (130–186)	143.5 (106–194)	75	0.144	100	0.109	67	0.285
Object insertion	630 (456–935)	392 (351–600)	250 (235–322)	270 (225–340)	75	0.144	100	0.102	67	1.00
Meniscus resection	364 (203–640)	204 (178–249)	82 (76–270)	94 (37–118)	100	0.068	67	0.285	67	0.285

No difference was noted in terms of time for task completion in the clear dome but under the opaque dome lesser experienced groups showed greater times. Significant difference was seen on comparison of performance between the groups only under the opaque dome where use of the scope was required (Table 4).

### Individual task results (Tables 3, 4 and Figures 2, 3)

1. Maze navigation: The mean time to completion under the opaque dome was sequentially less as experience level increased with Novice = 179.7 s (±71.4), Beginner = 130 s (±32.0), Intermediate = 104.3 s (±18.8) and Advance = 100.7 s (±23.8), difference between groups being significant with *p* = 0.027. Although oncomparing the improvement in performance (Table 4) between the groups, we found that there was no statistical significance (*p* > 0.05) between subsequent groups in terms of improvement of performance. However, it was notable that improvement was least between intermediate and advance groups (33% positive performance compared to 75% between novice and beginner and 67% between beginner and intermediate).
2. Number probing: As for the maze navigation task no significant difference was found under the TD but the increased experience groups were noted to have faster times under the OD. The mean times for each group under opaque dome were Novice = 657 s (±195.7), Beginner = 333 s (±166.9), Intermediate = 267 s (±119.3) and Advance = 258.2 s (±71.3) with *p* = 0.001, although the trend of improved performance was best between novice and beginner groups (100% positive performance).
3. Object handling: Similar results were noted as the other tasks, with Novice and Beginners being significantly slower than Intermediate and Advance under the OD with *p* = 0.011 for object retrieval and *p* < 0.001 for object insertion. There was 67% positive performance between intermediate and advance on paired comparison with actually an increased mean time for advance group (276.2 ± 49.2 s) versus intermediate group (269 ± 46.5 s) in the object placement task.
4. Partial menisectomy: Figure 4 shows the individual times for each group taken for MM and LM partial menisectomy under TD and OD. The novice group took almost three times the time under opaque dome than under transparent dome for either MM or LM with a *p* = 0.008 whereas the intermediate and advance had hardly any time difference for either the dome or the site of meniscus (*p* = 0.197).

### Error rate, face validity and subjective assessment

The errors among the groups were, as expected, higher for lesser experienced participants. There were no errors observed for any group under the transparent dome. Under the opaque dome, the median number of errors in maze navigation was novice-2, beginner-2, intermediate-0 and advance-0 and in object handling it was novice-7, beginner-5, intermediate-4 and advance-2. There were four out of nine novices with inappropriate meniscus resection as compared to one in beginner group and none in intermediate or advance group. The median rating on a scale of 1–5 for smooth scope navigation, image tracking, triangulation, instrument handling and bimanual dexterity was 4 (poor) for novice, 2.5 (between not so good and good) for beginners and 1 (very good) for both intermediate and advance groups. The same was the response to difficulty experienced. All the participants agreed that this model represented basic skills of scope movement, simultaneous image tracking and instrument use, tissue handling and basic meniscus resection skills required for arthroscopy. Irrespective of experience levels, 85% (17) felt there was improvement in performance with subsequent tasks and 90% felt this model would be useful in arthroscopic skills development. Except the advance, all other participants (16 of 20) expressed desire to train on this model to improve their skills.

## Discussion

**Figure 3.**
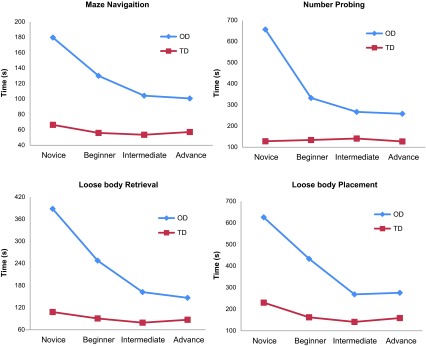
Graphical representation of mean time taken by groups to complete tasks under transparent dome (TD) and opaque dome (OD).

**Figure 4.**
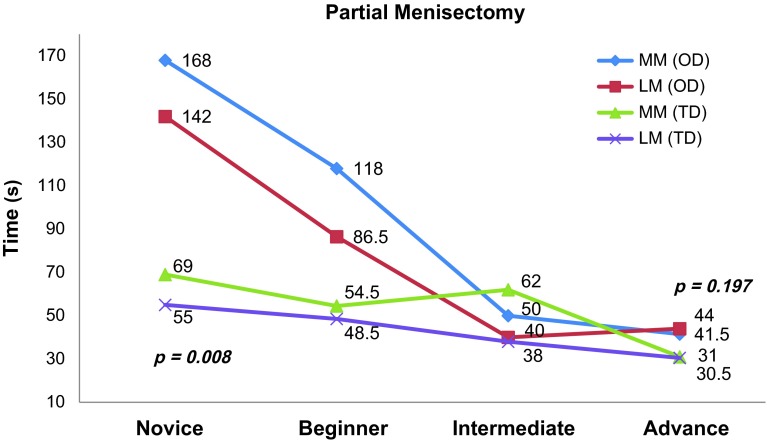
Individual times (s) for medial meniscus (MM) and lateral meniscus (LL) taken by groups under transparent dome (TD) and opaque dome (OD).

We observed that task performance under the transparent dome was not related to experience of the surgeon (Table 3, Figure 3) unlike the opaque dome which highlighted the importance of hand-eye coordination required in arthroscopy. Transparent dome tasks require isolated motor skills with direct visualization whereas the opaque dome requires visuo-spatial coordination which is a skill that develops with practice as shown by the difference in performance between the different groups of surgeons based on their experience. The times for maze navigation and number probing show no difference in performance between the groups although tasks for object handling and partial menisectomy show slight improvement in performance with increase in arthroscopy experience but it is insignificant (*p* > 0.05). When comparing the performance under the opaque dome we found that all the tasks are clearly able to distinguish the skills of surgeons based on experience (*p* < 0.05), suggesting the use of arthroscope and bimanual dexterity becomes more proficient as the experience increases. This is best observed on comparing the performance for partial menisectomy (Figure 4). We also observed that there is significant difference in time for novice and beginners between transparent and opaque domes (*p* < 0.005) but intermediate and advance level surgeons hardly had any difference (*p* = 0.197). This means that this model has the ability to distinguish between pure motor skills and the skills required while using an arthroscope. This establishes the construct validity, as it can effectively differentiate the surgeon's skills of using the arthroscope and instruments is different and require repeated practice for improving dexterity and visuo-spatial orientation.

When we compared arthroscopic skills between the groups, we focused on the time for task completion under the opaque dome as the surrogate for competency. There was a consistent trend for lesser time for completion with higher experience level (Table 3, Figure 3), except one aberration for the task of object placement where the advance took a mean of 276.2 s compared to 269 s when compared to intermediates. This difference between groups was statistically significant when comparing novice to all groups and beginner to intermediate and advance but not between intermediate and advance with highly significant difference (*p* < 0.005) for tasks like number probing, object placement and partial menisectomy. This establishes that fine difference in performance after a certain degree of experience requires more than just basic psychomotor skills.

These observations confirm our hypothesis that higher experienced arthroscopic surgeons will perform better in tasks requiring specific arthroscopic skills as seen in many previous studies [2, 7, 12, 15–17, 19, 22, 25–31]. We can thus suggest that this model has good construct validity. The differences between groups are not simply in mean time for task performance but also in performance change. The jump in improvement (Table 4) is seen most for novice to beginner (75–100%) followed by beginner to intermediate (67–100%) and least for intermediate to advance (33–67%). This improvement reflects that development of comprehensive technical skills in the early stages of arthroscopic training may be faster and more efficient. This kind of assessment would also allow identification of trainees who may pick up skills better than other. This is also shown in other studies where certain students acclimate to arthroscopy earlier than others [31, 32].

The face validity of this construct should be established before we can suggest its use as a surrogate for arthroscopic skills training. Using grading from very good to very poor to assess performance of different groups in terms of arthroscope navigation, image tracking, triangulation, object and instrument handling and bimanual dexterity we found that there was a progressive improvement in median grade from 4 (poor) to 1 (very good) from novice to advance. This was coupled by similar feedback response by participants as grade 4 (difficult) for novice to 1 (very easy) for advance group. Similar feedback assessment for face validity was used by Braman et al. [30]. We acknowledge that our analysis is limited by the fact that the number of participants in each of the groups was small and statistical comparison could not be made. Nonetheless, the objective construct validity was reinforced by the sequential improvement in performance and response-based face validity making the model applicable for training surrogate. The entire advance group with more than 15 years or arthroscopic experience between them agreed that these four tasks adequately represent the basic skills in arthroscopy.

Establishing benchmark criteria for objective assessment of skills and for a stepwise training is an important criterion for any training module. Our results show that, for novice to beginners and beginners to intermediates and/or advance, the time for task completion is significantly disparate and shows that the differences are in a gradual slope. We suggest the possibility of using the median time for task performance of beginners and intermediates as guideline to assess the improvement during training and for progression of skills in a structured program (from Table 4). If we take the task of meniscus resection as an example, a novice that begins training on day one and trains for a fixed period of time e.g. two weeks, then, to consider him competent with the technical skill, he should perform the task within 205 seconds (beginner level). At this stage the training continues and the next goal is to be able to do the same task within 80–95 seconds (intermediate-advance level). This would allow an objective assessment of skills and help trainers to recognize adequacy of skills in trainees to progress to clinical setting.

Cadavers and computerized high fidelity simulators which incorporate three-dimensional anatomy, virtual reality, haptic feedback, trajectory and force data analysis are useful in providing a training atmosphere which attempts to recreate anatomy, tissue response and clinical scenarios [2, 4, 7–9, 12–17, 21, 22, 26, 27, 33, 34]. Recent evidence also suggests that there is considerable ability to transfer skills acquired on simulators to the operating room [34]. However, both cadaveric and high technology computerized simulators are limited by availability, expense and resources [8, 9, 11, 19, 20, 35]. Low fidelity simulation allows opportunity to learn and practise basic skills with goal-directed modules; like the incentive to reach training benchmarks to allow trainees to progress [23, 24, 31, 32, 36]. We have used a low fidelity *box-type* bench model and assessed its utility in distinguishing arthroscopic skills and possibility of using the results to develop a structured training program.

### Limitations and future directions

This study is limited by small sample size and insufficient variation in tasks like more repetitions, changing orientation of platforms which would have provided more data for better comparison. However, we take this study as an opportunity to differentiate surgeons on basic skills and allow us to generate guidelines for training. We suggest using this or a similar module for assessing the baseline skills and then using the performance based on experience to develop a structured program. An example from this study that can provide a baseline to develop benchmark scores is timing of intermediate level surgeons. For the future, we aim to use these parameters for trainees on this model and assess their skills over a period of time (an on-going study). However long the time taken to reach the target score, the trainees need to stop when they reach intermediate level timings. Although further recommendations will not only depend on the time spent on the model but are influenced by other factors as well.

### Conclusions

Pure motor skills with direct visualization tasks are inherent skills of surgeon but the use of arthroscope needs visuo-spatial coordination; a skill that develops with practice as shown by the performance of different groups of surgeons based on their experience. From this study we conclude that this model has adequate construct validity for distinguishing level of basic arthroscopic skills among surgeons and provides us with guidelines for further research (which we are doing currently) to see progression of trainees as they spend more time training on this model. This model is especially useful in institutions where resources to a develop surgical skills laboratory are limited.

## Conflict of interest

The author(s) declare no conflict of interest in relation with this paper.
